# Electrochemical Nucleation and Growth in Battery Electrodes under Reactant-Limited Conditions

**DOI:** 10.1021/acs.nanolett.5c06068

**Published:** 2026-01-23

**Authors:** Jing Yu, Irina Martynova, Zeyan Li, Canhuang Li, Chaoqi Zhang, Qing Sun, Jordi Arbiol, Andreu Cabot

**Affiliations:** † Catalonia Institute for Energy Research (IREC), Sant Adrià de Besòs, Barcelona 08930, Catalonia, Spain; ‡ 231882 Catalan Institute of Nanoscience and Nanotechnology (ICN2), CSIC and BIST, Campus UAB, Bellaterra, Barcelona 08193, Catalonia, Spain; § State Key Laboratory of Tropic Ocean Engineering Materials and Materials Evaluation, School of Marine Technology and Equipment, Hainan University, Haikou 570228, China; ∥ College of Materials Science and Engineering, 12423 Fuzhou University, No. 2, Xueyuan Road, Minhou County, Fuzhou, Fujian 350108, China; ⊥ ICREA Pg. Lluis Companys, Barcelona 08010, Catalonia, Spain

**Keywords:** lithium−sulfur battery, electrocatalysis, electrochemical nucleation and growth

## Abstract

Nucleation and growth of solid phases from species dissolved in an electrolyte govern battery performance, defining capacity, efficiency, rate capability, stability, and safety. However, classical nucleation–growth models often do not realistically describe working cells, failing to capture highly asymmetric out-of-plane growth and finite reactant supply. Here, we introduce a nucleation–growth model to fit potentiostatic nucleation transients that explicitly accounts for a finite amount of reactant and its depletion, reproducing the characteristic current rise upon nucleation, peak, and subsequent decay without ad hoc corrections. Both instantaneous nucleation and progressive nucleation are considered. The model is applied to the nucleation and growth of Li_2_S at a catalyzed electrode from a lithium polysulfide solution, yielding nucleus densities of up to 6.7 × 10^9^ cm^–2^ and an effective reaction rate constant of 1.8 × 10^–3^ s^–1^. Beyond Li–S batteries, the framework can be extended to other conversion and metal-deposition chemistries in which finite-supply effects dominate.

## Graphical abstract

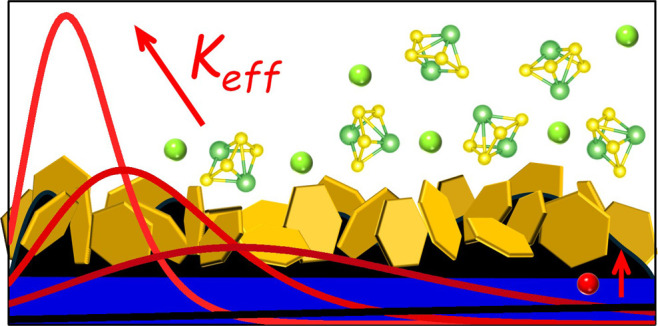

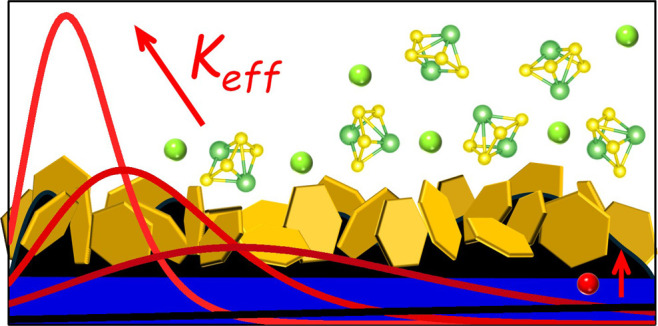

In some battery chemistries, the nucleation and growth of solid phases from species adsorbed on the electrode or dissolved in the electrolyte are fundamental in determining performance. These processes define deposit morphology and, in turn, govern capacity, overpotential, energy efficiency, rate capability, cycling stability, and even safety. Deposition of poorly conducting discharge products is, for example, a key kinetic bottleneck in conversion-type cathodes such as sulfur and oxygen, where the formation of electronically and ionically resistive phases imposes severe transport and interfacial limitations that ultimately cap practical capacity utilization, overpotential, rate performance, and cycling life.^1–4^ At metal anodes, nucleation and growth similarly dictate stability and safety. Lithium and zinc anodes, for instance, are prone to heterogeneous nucleation and anisotropic growth that amplify local electric fields and mass-transport gradients, leading to porous, mossy, or dendritic morphologies that accelerate side reactions, increase impedance, and, in the worst case, trigger short circuits.^5–9^

Nucleation and growth mechanisms in electrochemical systems are commonly probed using potentiostatic techniques, where the resulting current–time transient exhibits a characteristic peak that reflects the temporal evolution of the reaction as nuclei form and grow. Initially, the current increases as the reaction rate rises with the expansion of the electrochemically active surface area, driven by the formation of new nuclei and the growth of existing ones. As the diffusion zones surrounding individual nuclei begin to overlap, mass transport to the electrode becomes increasingly hindered, the current reaches a maximum, and then decays as growth becomes limited by reactant diffusion in the electrolyte.^10–12^

This behavior has been described mathematically by classical models such as those of Bewick, Fleischmann, and Thirsk (BFT) and later Scharifker and Hills (SH), which relate the normalized current transients to the dimensionality of growth (2D or 3D) and the nucleation mode (instantaneous or progressive).^13–15^ In these frameworks, nuclei are treated as electrically conductive domains anchored to the electrode surface that grow as laterally spreading films (2D) or hemispherical caps (3D) under uniform electric fields, with constant reaction rates at their surfaces and diffusion layers evolving in an otherwise unperturbed electrolyte.

These electrochemical nucleation–growth models, widely used to interpret the potentiostatic transients in battery research, often fall short in battery environments because they neither capture the growth of strongly asymmetric out-of-plane structures such as dendrites and platelets, nor account for the finite reactant supply of real working cells and the rapid local depletion characteristic of porous electrodes under potentiostatic or galvanostatic operation.

In real batteries, beyond the limited supply of reactant, the reaction can also be constrained by the higher overpotential required as insulating layers grow and increase impedance, and as the reactant concentration decreases according to the Nernst equation.

Here we introduce a nucleation–growth model that explicitly accounts for a finite reactant reservoir and its spatiotemporal depletion under potentiostatic operation, reproducing the characteristic current rise, peak, and decay. We apply this framework to Li_2_S deposition from a lithium polysulfide (LiPS) solution in Li–S cells, enabling robust fitting of chronoamperometric transients and quantitative assessment of the catalyst’s impact on nucleation and growth. In doing so, the model provides a more faithful link between experiment and mechanism, improving our ability to determine how catalysts, electrolytes, and interfaces jointly govern nucleation and growth in Li–S systems and, more broadly, in conversion and metal-deposition battery chemistries.

The electrodes investigated are based on a Co–Bi double-atom catalyst (DAC) supported on a carbon nitride (CN) framework (Co–Bi/CN, Figure 1a),^16^ a nanostructured MoS_2_ catalyst (Figure 1b), and bare CN (Figure 1c). Details of catalyst synthesis and electrochemical testing are provided in the Supporting Information. The Li_2_S deposit morphologies obtained after potentiostatic nucleation test at 2.05 V are shown in Figure 1d–f, corresponding to the current–time transients in Figure 1g. Representative galvanostatic charge–discharge (GCD) profiles of the three electrodes at 0.1C rate are presented in Figure 1h.

**Figure 1:**
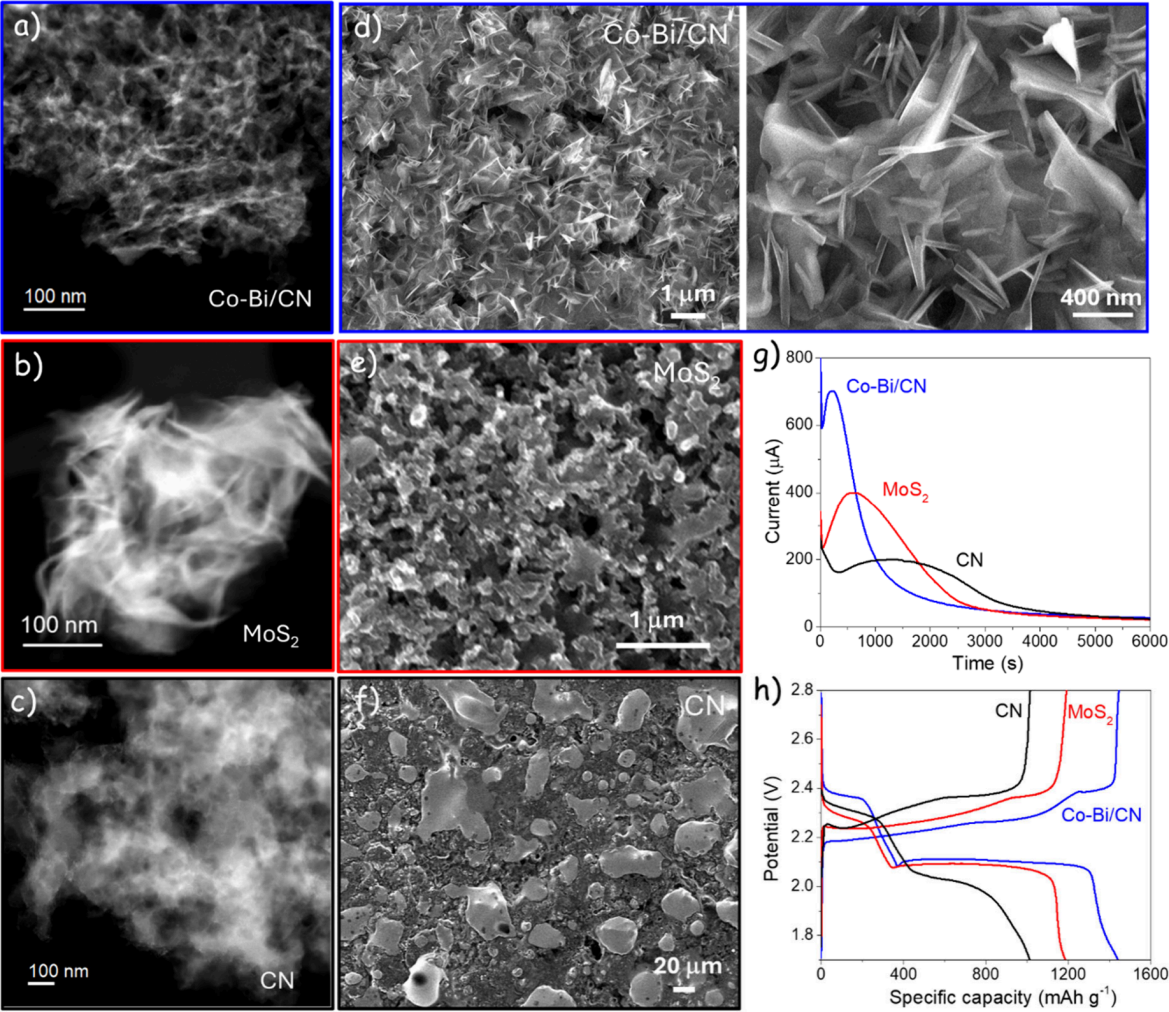
Li_2_S nucleation and growth on catalyzed electrodes. (a-c) High-angle annular dark field scanning transmission electron microscopy images of the electrode materials Co–Bi/CN, MoS_2_, and CN, respectively. (d–f) Scanning electron microscopy images of the Li_2_S particles obtained after potentiostatic nucleation tests on Co–Bi/CN-, MoS_2_-, and CN-based electrodes, respectively. (g) Potentiostatic nucleation transients at 2.05 V for the three electrodes. (h) GCD curves of Li–S coin cells based on the three electrodes.

Several features are inconsistent with the classical nucleation–growth models commonly used in battery studies, which assume 2*D*/3D diffusion-limited growth fed by an effectively infinite reactant reservoir. First, the integrated charge under the nucleation peaks is relatively similar across electrodes, independently of the produced Li_2_S morphologies. This is consistent with the fact that, given sufficiently long times and high overpotentials, low S_8_ loading cells can reach close to full S_8_–Li_2_S capacity disregarding of the catalyst. These observations argue against a dominant geometric blocking mechanism in which morphology and coverage alone determine the integrated current.

Second, for the Co–Bi/CN catalyst, the Li_2_S deposit forms a porous layer populated by highly asymmetric, plate-like particles oriented predominantly normal to the electrode plane. Such morphologies are not captured by standard models based on laterally spreading 2D islands or homogeneously growing 3D hemispherical domains.

Notice also that, in the conventional coin-cell configuration used, the separator that sets the anode–cathode distance, and thus the volume of the electrolyte reservoir, is just 25 μm thick and contains 20–40 μL of electrolyte. Taking an initial LiPS concentration of C = 0.25 M and a typical nonlean electrolyte diffusivity of D ∼ 10^–6^ cm^2^ s^–1^ at 25 °C, the root-mean-square diffusion length ( $L=\sqrt{2Dt}$ ) is already ∼ 10 μm, comparable to the separator thickness. A simple diffusion zone estimate from the classical model ( $r\left(t\right)=\sqrt{2DCMt/\rho }$ ) using a molar mass (M) of the electroactive species (e.g., Li_2_S_4_) of 142 g mol^–1^ and a Li_2_S density (ρ) of 1.66 g cm^–3^, gives *r*∼20 *μm*, similar to the separator thickness, after only 100 s of reaction, which is just a small fraction of the total nucleation and growth time. These length scales are clearly incompatible with the infinite-reservoir idealization implicit in classical models.

Overall, it is therefore not surprising that direct fits of the standard 2D and 3D models fail to accurately reproduce the full transient. This mismatch is often rationalized by invoking ad hoc switches between instantaneous and progressive nucleation or between 2D and 3D growth within a single experiment, assumptions that are likely far from the actual behavior.

As a result, the current peaks observed in batteries cannot be rigorously interpreted using classical electrochemical models, because the system no longer satisfies their underlying boundary conditions and simplifying assumptions. Applying these models outside their domain of validity can lead to oversimplified or even misleading conclusions about the nucleation and growth mechanisms governing deposition of solid products in battery electrodes. For example, a common mistake is to associate 2D growth models with the formation of plate-like structures extending out of the electrode plane, which is a clear misinterpretation of the original 2D model that actually describes lateral growth of islands confined to the electrode surface.

Taken together, these observations motivate a framework that explicitly includes finite reactant supply and depletion to better fit the entire potentiostatic transient with physically interpretable parameters without resorting to ad-hoc switching between classical models.

Given that further Li_2_S precipitation is more favorable on existing Li_2_S (homogeneous) than on the bare substrate (heterogeneous), we consider that the reaction rate, and thus the current, increases once nucleation begins and the surface area of Li_2_S available for further Li_2_S growth expands. At some point, however, the current starts to decrease not due to overlapping diffusion zones, since the entire electrolyte in the cell is already under depletion conditions, but because of the finite amount of reactant in the cell. This limited reservoir primarily constrains the total amount of material that can be converted and, secondarily, increases the overpotential required for the reaction to proceed, as dictated by the Nernst equation. In addition, the increasing thickness of the Li_2_S layer can further raise the overpotential owing to its moderate electrical conductivity. Thus, as the reactant concentration continues to drop while solid Li_2_S is deposited, the reaction rate and the current progressively slow down.

We assume that the growth rate of each new-phase particle is proportional to its surface area (*A*), and that the surface reaction rate (*J*) is directly proportional to the reactant concentration (*C*), i.e., a first-order reaction:

1 $$\frac{dV}{dt}=\Omega \frac{dm}{dt}=\Omega AJ$$

2 $$J=k_{s}C$$

where *V* is the volume of the growing solid particle, Ω is the molar volume of the new phase, $\frac{dm}{dt}$ is the reactant consumption rate, and *k*_*s*_ is the surface growth constant. Assuming spherical particles:

3 $$\frac{dV}{dt}=4\pi r^{2}\frac{dr}{dt}$$

From eq 1

4 $$\frac{dV}{dt}=\Omega 4\pi r^{2}k_{s}C$$

Combining eqs 3 and 4

5 $$\frac{dr}{dt}=\Omega k_{s}C$$

As the reaction proceeds at the particle surface, the overall monomer concentration in the electrolyte decreases. For instantaneous nucleation at time *t*_0_ with a nuclei density *N = N*_0_:

6 $$\frac{dN}{dt}=N_{0}\delta \left(t-t_{0}\right)$$

Further assuming nucleation at *t*_0_ = 0:

7 $$\frac{dC}{dt}=-\frac{N_{0}AJ}{V_{e}}=-\frac{4\pi r^{2}{N_{0}k}_{s}}{V_{e}}C$$

where *V*_*e*_ is the volume of electrolyte within the cell. Combining with eq 5

8 $$\frac{dC}{dr}=\frac{4\pi N_{0}}{\Omega V_{e}}r^{2}$$

Thus

9 $$C\left(r\right)=C_{0}-\frac{4\pi N_{0}}{3\Omega V_{e}}\left(r^{3}-r_{0}^{3}\right)$$

Then the current density as a function of the particle radius can be expressed as

10 $$i\left(r\right)=nFN_{0}AJ=nF4\pi N_{0}k_{s}r^{2}C\left(r\right)=nF4\pi N_{0}k_{s}r^{2}\left[C_{0}-\frac{4\pi N_{0}}{3\Omega V_{e}}\left(r^{3}-r_{0}^{3}\right)\right]$$

where *n* is the charge transferred in each surface reaction of the reactant and *F* is the Faraday constant. Assuming the initial particle radius is zero and defining the final particle radius (*r*_*f*_), a dimensionless radius (*y*), a dimensionless time (θ), and an effective rate constant (*k*_*eff*_) as

11 $$r_{f}\equiv {\left(\frac{{3\Omega V_{e}C}_{0}}{4\pi N_{0}}\right)}^{1/3}$$

12 $$y\equiv \frac{r}{r_{f}}$$

13 $$\eta \equiv \frac{\Omega k_{s}C_{0}}{r_{f}}t\equiv k_{eff}t$$

then, *y*(θ) is implicitly given by

14 $$\theta =-\frac{1}{3}\ln\left(1-y\right)+\frac{1}{6}\ln\left(1+y+y^{2}\right)+\frac{1}{\sqrt{3}}\left[\arctan\left(\frac{2y+1}{\sqrt{3}}\right)-\frac{\pi }{3}\right]$$

and the current density is

15 $$i\left(t\right)=nF4\pi N_{0}k_{s}C_{0}r_{f}^{2}y^{2}\left(\eta \right)\left(1-y^{3}\left(\eta \right)\right)$$

This expression yields the conventional peak-shaped transient (Figure 2a) and can be readily fitted to the experimental data to extract the time-scaling parameter *k*_*eff*_.

**Figure 2:**
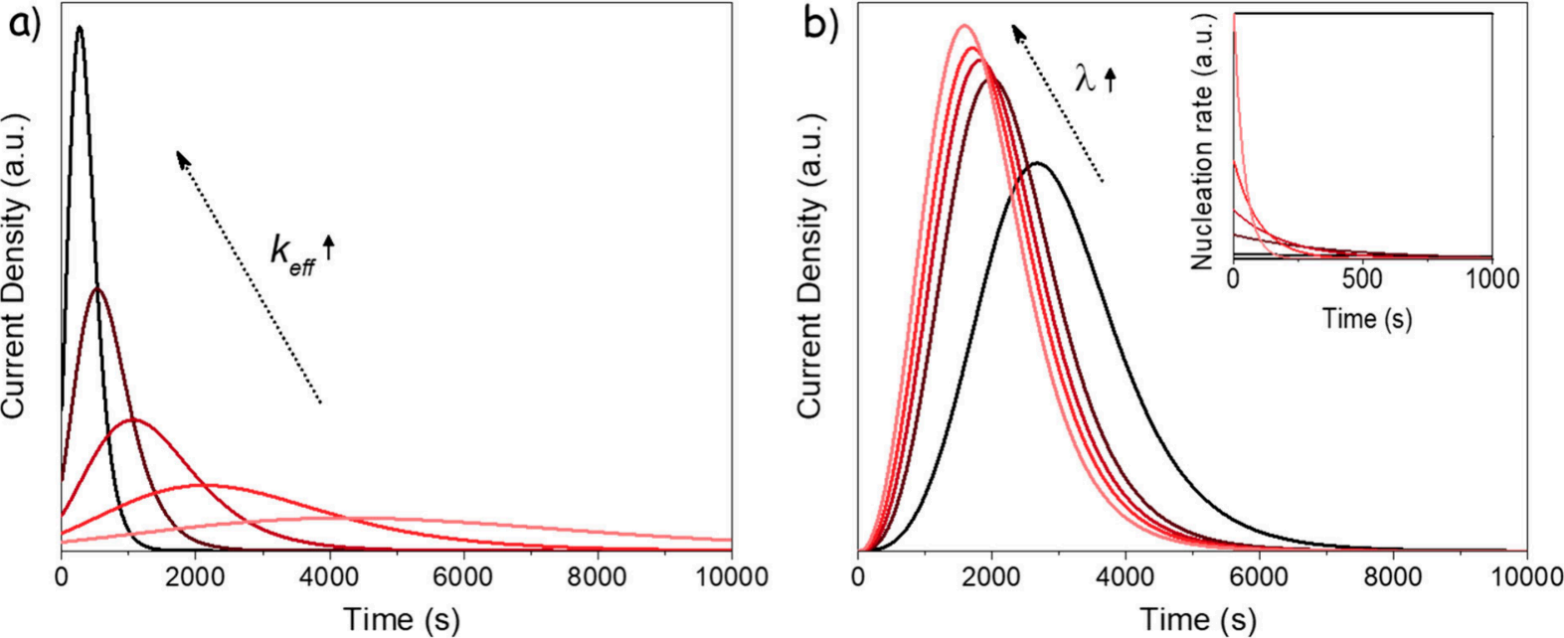
Simulated nucleation curves. (a) Instantaneous nucleation with *k*_eff_ values of 2 × 10^–3^, 1 × 10^–3^, 5 × 10^–4^, 2.5 × 10^–4^, and 1.25 × 10^–4^. (b) Progressive nucleation with a *k*_eff_ of 5 × 10^–4^, and λ values of 1, 5, 10, 20, and 50. The inset shows the nucleation rate as a function of time.

A similar expression is obtained when considering the growth of circular plates rather than spherical particles when we model each nucleus as a platelet of thickness *h* that increases proportionally to its radius (*h = ar*). To account for distinct growth kinetics at the basal planes and at the edges, we define an effective surface rate constant as *k*_*s*_ *=ak*_*se*_ *+k*_*sl*_ */2*, where *k*_*sl*_ and *k*_*se*_ are the growth constants at the edge and basal (lateral) planes, respectively:

16 $$\frac{dV}{dt}=3\pi ar^{2}\frac{dr}{dt}$$

17 $$\frac{dV}{dt}=\Omega 2\pi ar^{2}k_{s}C$$

18 $$\frac{dr}{dt}=\frac{2\Omega k_{s}}{3}C$$

Assuming instantaneous nucleation at *t*_0_ = 0:

19 $$\frac{dC}{dt}=-\frac{2\pi ar^{2}{N_{0}k}_{s}}{V_{e}}C$$

20 $$\frac{dC}{dr}=\frac{3\pi aN_{0}}{\Omega V_{e}}r^{2}$$

Considering initial nuclei with zero radius, we obtain a similar expression for *C*(*r*) and thus for *i*(*t*)­

21 $$C\left(r\right)=C_{0}-\frac{\pi aN_{0}}{\Omega V_{e}}r^{3}$$

22 $$i\left(t\right)=nF2\pi aN_{0}k_{s}C_{0}r_{f}^{2}y^{2}\left(\eta \right)\left(1-y^{3}\left(\eta \right)\right)$$

but now with

23 $$r_{f}\equiv {\left(\frac{{\Omega V_{e}C}_{0}}{\pi {aN}_{0}}\right)}^{1/3}$$

and a dimensionless time:

24 $$\eta \equiv \frac{2\Omega k_{s}C_{0}}{3ar_{f}}t\equiv k_{eff}t$$

Let us consider now a progressive nucleation with a nucleation rate, *P*(*t*), that is proportional to the available nucleation sites:

25 $$P\left(t\right)\equiv \frac{dN}{dt}=b\left(N_{0}-N\left(t\right)\right)$$

26 $$N\left(t\right)=N_{0}\left(1-e^{-bt}\right)$$

27 $$P\left(t\right)=N_{0}e^{-bt}$$

Now, each nucleus appearing at time τ grows according to the same growth law as before, i.e. reaction-limited by the reactant concentration and area proportional. Considering the growth of spherical particles:

28 $$\frac{dr\left(\tau ,t\right)}{dt}=\Omega k_{s}C\left(t\right)$$

The particle radius at each time *t* (*t < τ*) is

29 $$r\left(\tau ,t\right)=\Omega k_{s}\int_{\tau }^{t}C\left(u\right)du$$

The total volume of the new solid phase *V*_*s*_ *(t)* is

30 $$V_{s}\left(t\right)=\int_{0}^{t}P\left(\tau \right)\frac{4\pi }{3}{r\left(\tau ,t\right)}^{3}d\tau$$

The mass balance in the cell gives

31 $$C\left(t\right)=C_{0}-\frac{V_{s}\left(t\right)}{\Omega V_{e}}=C_{0}-\frac{4\pi }{3\Omega V_{e}}\int_{0}^{t}P\left(\tau \right){r\left(\tau ,t\right)}^{3}d\tau$$

Then the total current density is

32 $$i\left(t\right)=nF4\pi k_{s}C\left(t\right)\int_{0}^{t}P\left(\tau \right){r\left(\tau ,t\right)}^{2}d\tau$$

Considering the (maximum) final particle radius and the dimensionless time defined in eqs 11 and 13, we define a dimensionless radius of each particle that now depends on its nucleation time, *y*(τ, *t*), a dimensionless concentration *c­(η)*, an integrated dimensionless concentration *U­(η)*, and a dimensionless nucleation rate parameter (λ):

33 $$y\left(\tau ,t\right)\equiv \frac{r\left(\tau ,t\right)}{r_{f}}$$

34 $$c\left(\eta \right)\equiv \frac{C\left(t\right)}{C_{0}}$$

35 $$U\left(\eta \right)\equiv \int_{0}^{\eta }c\left(\xi \right)d\xi$$

36 $$\lambda \equiv \frac{br_{f}}{{\Omega k_{s}C}_{0}}$$

The radius of a nucleus born at η_τ_ and observed at η becomes

37 $$r\left(\tau ,t\right)=r_{f}\left[U\left(\eta \right)-U\left(\eta_{\tau }\right)\right]$$

From eq 33

38 $$y\left(\tau ,t\right)=U\left(\eta \right)-U\left(\eta_{\tau }\right)$$

and from eq 27

39 $$P\left(t\right)dt=dN=N_{0}\lambda e^{-\lambda \eta_{\tau }}d\eta_{\tau }$$

Then

40 $$c\left(\eta \right)=1-\lambda \int_{0}^{\eta }e^{-\lambda \xi }{\left[U\left(\eta \right)-U\left(\xi \right)\right]}^{3}d\xi$$

Then the dimensionless current density *j­(η)* is (Figure 2b):

41 $$j\left(\eta \right)\equiv \frac{i\left(\eta \right)}{i_{0}}=c\left(\eta \right)\lambda \int_{0}^{\eta }e^{-\lambda \xi }{\left[U\left(\eta \right)-U\left(\xi \right)\right]}^{3}d\xi$$

Figure 3 shows the potentiostatic transients fitted with both instantaneous and progressive nucleation models, incorporating an additional exponential term to account a priori for the initial double-layer charging decay.

**Figure 3:**
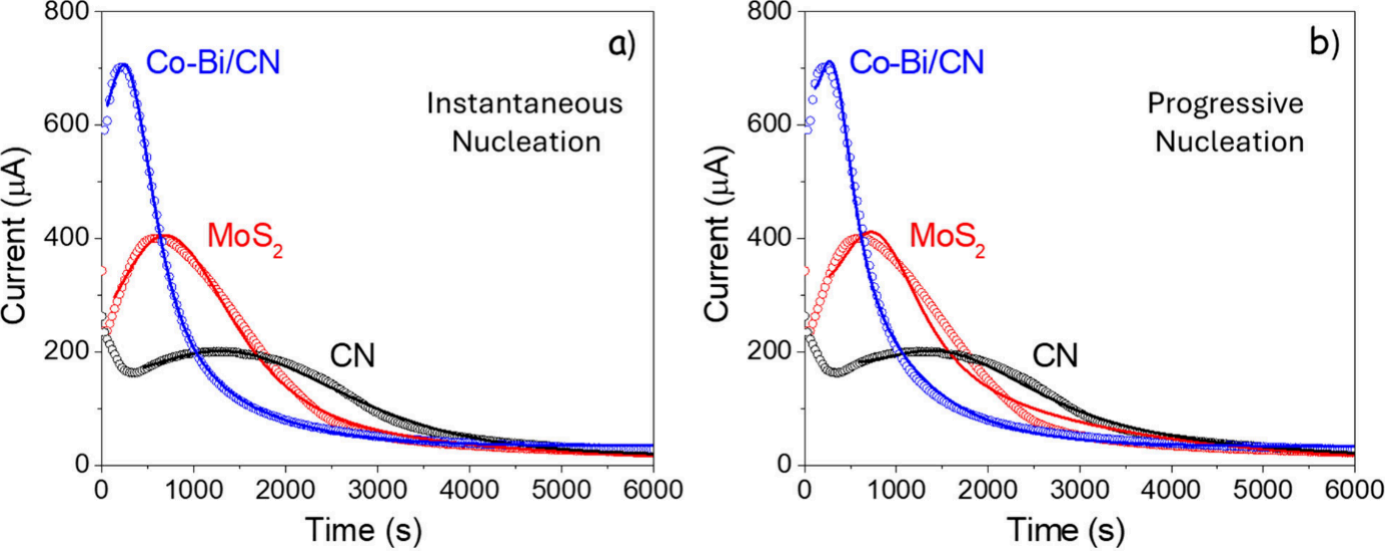
Fitted potentiostatic curves. (a and b) Potentiostatic nucleation transients at 2.05 V for the three electrodes fitted with the instantaneous and progressive nucleation models, respectively.

The progressive nucleation model does not improve on the results obtained with the simplified instantaneous nucleation assumption. In the best progressive fit, the nucleation rate decays so quickly that nucleation is only significant during the first ∼ 100 s, a small portion of the transient that overlaps with the initial exponential decay, which is itself not well captured by the fit. In this regime, a large nucleation constant (λ) makes progressive nucleation effectively equivalent to an instantaneous burst, as the nuclei population saturates much faster than particle growth and reactant depletion, which mainly determine the peak shape. We therefore rely on the instantaneous nucleation model in the following discussion.

The resulting fits are reasonably accurate, with R^2^ values above 0.99 for all electrodes. However, achieving this agreement requires relatively long initial exponential decays, particularly for the CN sample, which are not compatible with a conventional double-layer charging contribution; we will return to this point below. With this additional phenomenological term, outside the scope of the simple nucleation–growth model presented, the extracted effective rate constants, K_eff_, are 1.8 × 10^–3^, 7.0 × 10^–4^, and 3.7 × 10^–4^ s^–1^ for Co–Bi/CN, MoS_2_, and CN, respectively, in line with their relative catalytic activities.

The Co–Bi/CN electrode yields Li_2_S nanoplates with an average diameter of ∼ 1 μm and an average thickness of ∼ 40 nm (Figure 1d). Using the mass balance given by eq 23 and assuming complete conversion of the initial LiPS, we obtain a nuclei (particle) density of approximately 6.7 × 10^9^ cm^–2^. From the integration of the current–time curve and the corresponding charge conversion, a consistent particle density of 5 × 10^9^ cm^–2^ is obtained. Combining this nuclei density with the *k*_*eff*_ obtained from the fitting of the instantaneous nucleation model, we determine a surface growth constant of 130 nm s^–1^, which involves a plate radius growth speed of 0.9 nm s^–1^.

For the other electrodes, the Li_2_S particle geometry is less well-defined, which prevents a reliable direct determination of the nuclei density. These ill-defined morphologies likely arise from nucleation of Li_2_S domains with poorer crystallographic order. Such more amorphous-like particles may provide additional defect sites for subsequent homogeneous nucleation, leading to smaller apparent particle sizes.

We assume the catalyst mainly promotes the Li_2_S nucleation. After nuclei form and spread, the catalyst surface becomes progressively covered, and growth should proceed predominantly at the Li_2_S/electrolyte interface. In that regime, the relevant interfacial chemistry and charge transfer are no longer directly affected by the underlying catalyst, so it is reasonable to assume that the surface growth constant *k*_*s*_ does not vary significantly between samples. The catalysts could, in principle, affect soluble intermediate speciation/activation and the crystallinity of the precipitate, which could modify *k*_*s*_. However, introducing large *k*_*s*_ differences leads to inconsistencies: because *k*_*eff*_ is highly sensitive to *k*_*s*_, even moderate increases in *k*_*s*_ imply much larger *k*_*eff*_ variations than observed unless compensated by unrealistically low nucleation rates for the more active catalyst, which is not realistic. For this reason, we adopt a common *k*_*s*_ as a conservative working assumption and attribute the observed differences primarily to variations in nucleation behavior. Under this assumption, the fitted *k*_*eff*_ values yield a nuclei density for MoS_2_ that is 16 times lower than for Co–Bi/CN, i.e., 4.2 × 10^8^ cm^–2^, while the CN electrode exhibits a nuclei density 2 orders of magnitude lower than Co–Bi/NC, at 6 × 10^7^ cm^–2^.

We note that the fit for the Co–Bi/CN curve is considerably better. The poorer agreement for MoS_2_ and CN reflects the stronger asymmetry of their peaks, which necessitates longer exponential decay components in the fit and correlates with the smallest Li_2_S particle sizes observed for these electrodes (Figure 1e,f). In these cases, the growing particles strongly overlap, so beyond a certain point, the effective Li_2_S surface area available for further homogeneous monomer reaction no longer increases with reactant consumption as assumed in the ideal model and may even start to decrease. This overlap slows the decay of the current, yielding broader peaks with a more gradual decline than the symmetric peak shape predicted by the simple instantaneous nucleation-and-growth model described above. On the other hand, the Co–Bi/CN sample grows a more porous layer of plates with a reduced overlap, resulting in a more reproducible peak decay.

Overlap of the growing domains can be modeled by treating them as randomly distributed hemispherical caps whose superposition follows Poisson statistics, which yields an effective reaction area (*A*_*eff*_) of

42 $$A_{eff}\left(t\right)=A_{0}\theta \left(t\right)+A_{NO}\left(t\right)\left[1‐\theta \left(t\right)\right]$$

where *A*_0_ is the electrode/carbon area on which the new phase nucleates and grows, *A*_*NO*_ is the hypothetical active area in the absence of overlap, and θ is the surface coverage given by

43 $$\theta \left(t\right)=1-e^{-Y\left(t\right)}$$

where

44 $$Y\left(t\right)\equiv \frac{N_{o}\pi {R\left(t\right)}^{2}}{A_{0}}$$

Figure 4a shows the theoretical nucleation curves obtained at different overlapping strengths given by ϕ, which depends on the number of generated nuclei, the amount of reactant, the geometry of the growing particles, and the available area for the nucleation of the new phase.

45 $$\phi \equiv {\left(N_{o}\pi \right)}^{1/3}/A_{0}{\left(\frac{3}{2}\Omega C_{0}V_{e}\right)}^{2/3}$$

**Figure 4:**
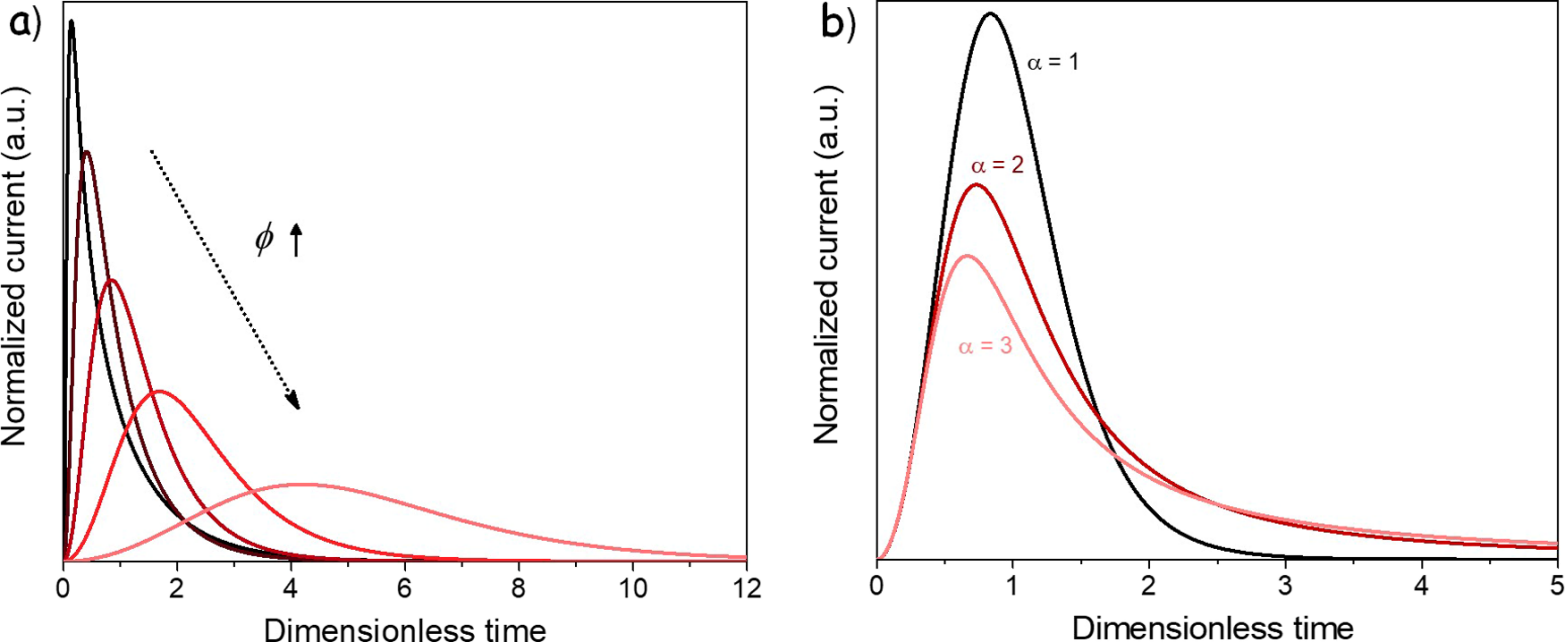
Simulated asymmetric nucleation curves. (a) Reactant depletion model, including overlapping with different strengths (ϕ = 0.2, 0.5, 1, 2, and 5). (b) Model considering different reaction orders C^α^ with α = 1, 2, and 3.

Beyond phase overlapping, additional factors may also contribute to the asymmetric shape of the experimental transients. One is the roughness of the carbon surface on which Li_2_S nucleates, which can create spatially inhomogeneous surface potentials and therefore nonuniform nucleation and growth kinetics, leading to asymmetric current profiles. This effect is difficult to capture within the present model; therefore, measurements on flatter substrates should be considered to obtain cleaner transients that are more suitable for quantitative fitting.

Another factor is the effective reaction order with respect to the reactant concentration. In our model, we considered a first-order dependence, which is a conventional assumption, but the complex sequence of processes in Li–S cells could lead to lower or higher apparent reaction orders and thus modify the peak shape. Lower orders would produce a slower current rise and a faster decay, which is not observed, whereas higher orders slow down the decay and broaden the peak, in line with our data (Figure 4b).

An additional potential asymmetry factor is the presence of concatenated electrochemical steps. Multiple peaks are frequently observed and can originate from additional redox transitions, for example, associated with incomplete prior discharge of S_8_ or Li_2_S_8_ to the Li_2_S-forming polysulfide monomer.^4^ In this context, the broad peaks obtained for the MoS_2_ and CN catalysts could reflect a two-step pathway, e.g., initial Li_2_S_2_ formation followed by its nucleation into Li_2_S. If the second step is slow, this would naturally generate an extended tail.

A further possibility is the involvement of chemical reactions that sustain Li_2_S growth once the domains become sufficiently large that charge transfer through them is strongly hindered. In this scenario, LiPS could be electrochemically activated at the bare surface and then chemically disproportionate to Li_2_S/Li_2_S_2_ at the Li_2_S interface, acting as a redox mediator themselves. If limited by LiPS activation, this process would slow down not only as LiPS concentration decreases but also as the free surface area shrinks, leading to a long tail as the available bare surface asymptotically approaches zero. A similar long tail would be expected if chemical disproportionation itself becomes rate-limiting and gains relative weight in Li_2_S formation as particles grow and both charge-transport pathways and the density of carbon–electrolyte–Li_2_S triple points decrease.

Overall, while the model reproduces the main nucleation–growth peak reasonably well, Li–S conversion involves a cascade of coupled electrochemical and chemical steps that cannot be fully captured by such a simplified framework. It does not account for the slow exponential decays required in the fitting, particularly evident for the CN sample, nor for the complex behavior of less active catalysts, where amorphous Li_2_S, particle overlap, and extensive polynucleation give rise to poorly defined morphologies. In addition, potentiostatic nucleation experiments are intrinsically sensitive to experimental details such as sample history, the rate at which the nucleation potential is approached, the exact nucleation potential, the total amount of reactant, and the support architecture. Nevertheless, by explicitly incorporating a finite reactant supply, the present model provides a more realistic description of the nucleation and growth of solid discharge products in batteries than classical electrochemical models that neglect the specific constraints of working cells, and offers a solid foundation on which more comprehensive descriptions of Li–S conversion and other complex systems can be built.

## Supplementary Material



## Notes

Additional experimental details about the synthesis of the materials and the electrode preparation, cell assembly, and nucleation tests (PDF)
