# Supplementary material for: Electrochemical Nucleation and Growth in Battery Electrodes under Reactant-Limited Conditions
Source: Nano Lett. 2026 Jan 23;26(4):1534–41. doi: 10.1021/acs.nanolett.5c06068 (PMC12879943; doi:10.1021/acs.nanolett.5c06068)
Supplement: Supplementary file 1 [file nl5c06068_si_001.pdf]

SUPPORTING INFORMATION

Electrochemical Nucleation and Growth in Battery Electrodes Under Reactant-Limited Conditions

Jing Yu[1,2], Irina Martynova[1], Zeyan Li[1], Canhuang Li[3], Chaoqi Zhang[4], Qing Sun[1], Jordi Arbiol[2,5], Andreu Cabot[1,5,*]

1 Catalonia Institute for Energy Research (IREC), Sant Adrià de Besòs, Barcelona 08930, Catalonia, Spain.

2 Catalan Institute of Nanoscience and Nanotechnology (ICN2), CSIC and BIST, Campus UAB, Bellaterra, 08193 Barcelona, Catalonia, Spain.

3 State Key Laboratory of Tropic Ocean Engineering Materials and Materials Evaluation, School of Marine Technology and Equipment, Hainan University, Haikou 570228, China.

4 College of Materials Science and Engineering, Fuzhou University, No.2, Xueyuan Road, Minhou County, Fuzhou City, Fujian Province 350108, China.

5 ICREA Pg. Lluis Companys, Barcelona 08010, Catalonia, Spain.

*Corresponding author: acabot@irec.cat

Experimental details

Synthesis of Co-Bi/CN and CN: Chloroanilic acid (417.96 mg, 2 mmol) was placed into a three-necked round-bottom flask under an argon atmosphere, and the flask was cooled in an ice bath. N-methyl-2-pyrrolidone (NMP) (12 mL) was used as the solvent, and melamine (504.48 mg, 4 mmol), 20 mg $BiCl_3$, and the same amount of cobalt chloride ($CoCl_2$) were introduced into the mixture at around 0°C. While stirring vigorously, a few drops of concentrated sulfuric acid were slowly added. After 20 minutes of continuous stirring, the ice bath was removed, and the reaction mixture was allowed to warm to room temperature. The solution was then heated to 170 °C for 24 hours under an argon atmosphere. Upon cooling to room temperature, the mixture was vacuum-filtered, washed with ethanol and water three times, and freeze-dried for 24 hours. The resulting black solid was

annealed at 700 °C for 3 hours under an argon atmosphere, with a temperature ramp of 5 °C/min. CN was obtained following the same procedure, but introducing no metal chlorides.

Synthesis of MoS_2: Ammonium molybdate tetrahydrate (0.802 g, 0.65 mmol) was dissolved in 15.0 mL of deionised water. Thiourea (0.913 g, 12.0 mmol) was then added, and the mixture was stirred at room temperature for 1 hour. The solution was subsequently transferred to a Teflon liner inside a stainless steel autoclave and heated at 180 °C for 24 hours. The resulting product was recovered by centrifugation and washed three times with deionised water and isopropanol. Finally, the obtained powder was dried under vacuum at 60 °C.

Electrode preparation, cell assembly, and nucleation test: Different catalyst materials were first mixed and heated with sulfur at 155 °C for 12 h at a mass ratio of 3:7. The resulting composites were then mixed with Super P and PVDF at a mass ratio of 8:1:1 to prepare the cathode slurry, which was uniformly coated onto carbon-coated aluminum foil. After drying at 60 °C for 8 h, the electrodes were punched into 12 mm-diameter disks. Coin cells were assembled in an Ar-filled glove box using the prepared cathodes, 40 μL of electrolyte (1M LiTFSI, DOL/DME, 1% $LiNO_3$), and a lithium disk anode. After a 3 h rest period, the cells were discharged at 0.1C to 2.12 V, followed by a 2 s rest, and then subjected to potentiostatic discharge at 2.05 V. Subsequently, the cells were disassembled in the glove box. The cathodes were rinsed with two drops of DOL and dried before SEM characterization.
